# Trauma survivors’ perceptions and experiences of prolonged exposure for PTSD at a psychology clinic

**DOI:** 10.4102/sajpsychiatry.v29i0.1869

**Published:** 2023-02-27

**Authors:** Duane D. Booysen, Ashraf Kagee

**Affiliations:** 1 Department of Psychology, Faculty of Humanities, Rhodes University, Grahamstown, South Africa; 2 Department of Psychology, Faculty of Arts and Social Sciences, Stellenbosch University, Stellenbosch, South Africa

**Keywords:** Low-resource community, psychotherapy, post-traumatic stress disorder, prolonged exposure, trauma

## Abstract

**Background:**

Several trauma-focused treatments have been developed to treat post-traumatic stress disorder (PTSD). Yet there are limited studies on how trauma survivors perceive and experience trauma-focused treatments such as prolonged exposure therapy (PE) for PTSD, especially in low- and middle-income countries (LMIC).

**Aim:**

The study aimed to explore the perceptions and experiences of trauma survivors receiving prolonged exposure therapy for PTSD and the general acceptability of PE for PTSD in a LMIC.

**Setting:**

The study was conducted at a community psychology clinic in the Eastern Cape, South Africa.

**Method:**

Using a qualitative method, seven adult trauma survivors who completed six sessions of brief PE for PTSD were interviewed. Thematic analysis was used to identify relevant themes and to understand how participants perceived and experienced PE for PTSD.

**Results:**

The analysis yielded five themes, namely structure, obstacles, gender, exposure and experiences of recovery.

**Conclusion:**

The findings suggested that participants perceived and experienced PE to be generally beneficial for the treatment of PTSD. Moreover, the study suggested that PE is an acceptable trauma therapy in a contextually diverse setting such as the Eastern Cape, South Africa. Overall, considering the evidence base of PE for PTSD, this study contributed to the literature on the acceptability of PE in a South African setting.

**Contribution:**

The findings of the study are in keeping with the extant literature on how persons perceive and experience PE for PTSD. The findings of the study suggests that PE is an acceptable and beneficial trauma therapy for PTSD in a contextually diverse setting such as South Africa. It is recommended that large scale implementation studies be conducted to further investigate the effectiveness, feasibility, and acceptability of PE in South Africa.

## Introduction

The psychological treatment of post-traumatic stress disorder (PTSD) has gained much attention over the last three decades.^1,2^ Empirically supported trauma therapies have accrued substantial empirical evidence to demonstrate their effectiveness to improve symptoms of PTSD.^3^ Yet little is known about the implementation of prolonged exposure (PE) and similar trauma therapies in low- and middle-income countries (LMICs).

More broadly, global initiatives to develop and implement knowledge to promote equitable mental healthcare for common mental disorders have been described as ‘painfully slow’ (p. 1).^4^ Mental healthcare in LMICs is beleaguered by political instability, limited healthcare infrastructure and limited trained professionals, among others.^4^ As a result, the overall treatment gap for common mental disorders in LMICs is estimated to be as high as 93% and in South Africa as high as 85%, especially in low-resource communities.^5,6^

Yet alongside these systemic challenges, there is also emergent literature towards the advancement of effective and contextually responsive psychotherapies in LMICs.^7^ Studies on psychotherapy in LMICs have explored the feasibility of task-sharing approaches, transdiagnostic interventions and the use of implementation science to increase the uptake of psychotherapy in LMICs.^7^ For example, Rossouw et al.^2^ conducted a comparative randomised clinical trial that investigated the effectiveness of PE for adolescents (PE-A) compared to supportive counselling (SC) for PTSD in a group of school learners aged 13–18 years (*n* = 63) in the Western Cape, South Africa.^2^ Rossouw et al.^2^ found that PTSD symptom severity in the PE-A group was significantly greater than that in the SC group at post-treatment and at the 12- and 24-month follow-up time points.^8^ Besides the positive treatment outcomes in the Rossouw et al.^2^ study, qualitatively, the acceptability of PE in LMIC remained questionable.

Research on participant experiences of exposure-based treatments is limited. Hundt et al.^9^ explored the reasons for dropout in a sample of 27 American military veterans who received PE, the most significant of which were related to the therapy itself. Participants stated that they did not have a good understanding of the rationale for PE and experienced the therapeutic alliance as poor and the treatment as ‘too stressful’ (p. 9).^9^ To our knowledge, there is only one study that has explored how South African clients experienced PE for PTSD. Van de Water et al.^10^ studied a sample of adolescents (*n* = 10) and nonspecialist health workers (NSHWs) (registered nurses) (*n* = 3) from the Western Cape. These authors found that both learners and NSHWs reported that the calm breathing component and the therapeutic relationship were conducive to PTSD symptom reduction.^10^

In addition to effectiveness and implementation studies of trauma therapies, studies on how treatments are perceived and experienced by clients are also needed, especially in LMICs. In this study, we investigated how trauma survivors perceived and experienced brief PE for PTSD at a community psychology clinic in the Eastern Cape, South Africa.

## Research methods and design

This study was part of a larger research project that explored the effectiveness of PE in ameliorating symptoms of PTSD and how participants experienced the treatment. Participants were recruited via poster advertisements and referred from counselling centres at the respective university. Participants completed a baseline assessment to diagnose PTSD, using the PTSD Symptom Scale Interview (PSSI-5).^11^ Baseline assessments were conducted by the first author, who also implemented the treatment. Once the treatment was completed, participants were invited to share how they experienced PE therapy for PTSD.

### Case descriptions

Thembi, a 25-year-old student from Gauteng, started therapy four months after she was assaulted and stabbed with a knife whilst studying in a local park near the university campus. Thembi had a history of multiple sexual traumas; at age 5–11 years, she was sexually assaulted by an adult female friend of her family. At age 22, she was raped by a male friend inside her home. Consequently, Thembi reported having had experiences of depression, characterised by low self-esteem and interpersonal difficulty.

Fiona, a 20-year-old student from the Western Cape, was sexually assaulted and raped by a female cousin. At age 12, her uncle sexually assaulted her, but she narrowly escaped by calling for help. Her experience of sexual trauma was never spoken about in her family. As a young adult, Fiona tended to isolate herself and struggled to function socially or to engage in romantic relationships.

Olga, a 21-year-old student from Gauteng, was raped by a friend from university. Olga did not inform the police about the rape as she did not want her family to know about her trauma. Olga felt confused about the rape because the perpetrator was a friend and she still saw him on campus. As a result, she struggled academically and was avoidant of the perpetrator.

Thando, a 21-year-old student from Gauteng, was stalked by an unfamiliar man in town who had exposed his genitalia to her in public, which led her to seek safety. As a result, she felt afraid to go to town as she believed that the perpetrator would find her and possibly rape her. In addition, Thando reported that her male cousin sexually assaulted her from age 7 to 12. At age nine, she witnessed her father being shot and seriously injured, and at age 14 she was physically attacked with a knife. Thando had difficulty with depression and low self-esteem.

Buyiswa, a 20-year-old student from the Eastern Cape, reported that three unknown men sexually assaulted her. Three months post-trauma, Buyiswa started trauma therapy and reported that she could remember certain parts of the assault and was unsure of the extent of the assault. Buyiswa stated that she did not inform her mother and found difficult to receive support from her family, as she did not want to burden them.

Anna, a 27-year-old student, reported that as a child she witnessed prolonged interpersonal violence between her mother and father, and that her father was emotionally abusive. At age 16, she witnessed her mother being shot and killed during a home break-in. At age 22, she reported that a work colleague raped her, and she believed that he drugged her after having supper with him. Anna found it difficult to trust people and had conflicting feelings toward her estranged father.

Tessa, a 20-year-old student from KwaZulu-Natal, was raped at age 15 and again at age 18. Tessa reported that she struggled with depression and low self-esteem. She described her relationship with her parents as emotionally guarded. According to Tessa, her family did not know about her sexual traumas. Tessa found it difficult to talk about her past and current traumas and avoided emotionally overwhelming experiences.

### Data collection

An interview schedule to guide the semistructured interview process was used. Interview questions were developed and refined by the authors before the interviews were conducted. Interview questions were: (1) How did you experience PE compared to your previous experiences of counselling?; (2) How did you experience the way sessions were structured?; (3) How did you experience the procedures of imaginal exposure and *in vivo* exposure? and (4) How did you experience the therapist who treated you? All interviews were conducted by a research assistant to ensure that the participants could express themselves in a candid manner about the treatment experience.

### Procedure

Standard PE therapy consists of 10–15 sessions of 90 min.^1^ We implemented an abbreviated version of PE, consisting of six sessions of 60 min and the same components of standard PE, which were: psychoeducation; breathing training; imaginal exposure, which is the recounting of the trauma memory; and *in vivo* exposure, which is to approach safe situations that are avoided. Similar to standard PE, emotional processing was conducted to allow the participants to process thoughts and emotions that were significant during the exposure. A full discussion of the treatment is presented in Booysen & Kagee, 2021.^23^

### Data analysis

We used thematic analysis (TA) to analyse the textual data and to identify overarching themes. Data or sample saturation is not entirely consistent with the values and assumptions of TA, and the value of qualitative research is within the interpretive work and not the amount of data or sample size^13^ Thus, the sample size (*n* = 7) was deemed appropriate, which was supported by a detailed case description of each participant and an analysis of the textual data.^14^ Firstly, we read each transcript to gain familiarity with the data. In addition to the initial reading of the transcripts, audio-recorded interviews were also used to check the accuracy of the data. Secondly, through the initial reading process, we made initial notes to start generating codes. The coding process was based on the guidelines suggested by Braun and Clarke.^15^ Thirdly, once coding was completed, we started identifying themes within the data. We then reviewed the themes for relevance and accuracy and developed theme names to organise the data. To ensure trustworthiness of the data, complete records of the study process, data collection and data analysis were kept. The analysis process and writing of the results were also discussed by the authors so that the credibility of the study could be maintained.

### Ethical considerations

Ethical approval was obtained from the Human Research Ethics Committee of Rhodes University (reference number 9633672). All participants signed a consent form and received an information flyer about the treatment and interviews. Participants could decline and/or withdraw any time.

## Results

The analysis of the textual data produced five themes, which are presented below.

### Structure of prolonged exposure

Thando’s experience of PE was different to her previous experiences of psychotherapy. She stated, ‘I think I appreciated having that structure because the fact that there was a goal to get to, and that we would get to the goal was very helpful’. Thando’s statement emphasises her appreciation of knowing what the treatment entailed and working towards a particular goal. Similarly, Tessa reported that, ‘… I liked the fact that it focused on one thing rather than all my issues [*laughs*]’. Her reference to ‘all my issues’ suggests that she probably had several life stressors and that she appreciated the focus on a specific stressor in therapy. A structured approach might also have mitigated feelings of anxiety and may have provided the necessary support and emotional containment to process a specific stressor. Participants stated that they appreciated the clarity about the nature of the therapy and what was to be expected of them during treatment.

### Obstacles

For Tessa, thinking and talking about her sexual trauma proved to be challenging at times. She reflected on how she wanted the therapist to be more supportive when she spoke about her experience of rape: ‘I expected there to be, uhm … emphasis on “it wasn’t your fault”’. For Tessa, she wanted more reassurance and warmth from the therapist. Her experience was insightful as feedback pertaining to the more subtle and sometimes overlooked relational experiences of psychotherapy.

Anna, as a child, witnessed prolonged periods of interpersonal violence between her parents, and she was raped as a young adult. Anna was frustrated by the structure and singular focus on PTSD. She stated that she wanted ‘a one-sentence answer to cure my problem’. Anna appeared to have entered the treatment with an expectation of receiving a ‘cure’ that could resolve her experiences of trauma. Her experience showed not only how a structure facilitated a better understanding of therapy but also how the therapist needed to be flexible and responsive to the expectations and needs of the client.

### Gender

The following quotation highlights the potential complexities and effects of gender in trauma therapy:

Interviewer ‘How did you find it talking to Duane and this whole process?’

Tessa ‘Sometimes it was a little uncomfortable, I guess. Talking about my experiences with a man.’

For Tessa, talking to a therapist of the opposite sex about her experience of sexual trauma was challenging. Furthermore, Tessa continued:

Interviewer ‘What made it uncomfortable, if you do not mind me asking?’

Tessa ‘I did not know how much detail to go into. I think that is what made it uncomfortable.’

Interviewer ‘Did you feel … safe?’

Tessa ‘Yeah.’

This quote highlights not only the potential complexities of gender in trauma therapy, especially sexual trauma, but also the practical considerations that can occur in the case of the therapist and participant if their genders are different. For example, Tessa reported that she did not know how much detail to provide during therapy about her experience of rape, which led her to feeling uncomfortable. Even so, Tessa still reported a sense of safety, irrespective of not having clarity regarding how to disclose sensitive experiences. Moreover, the effect of gender difference in trauma therapy for sexual violence can have unforeseen challenges and opportunities. For example, a rape survivor might believe that she is unable to trust men due to her experience of being raped by a man. Yet working with a male therapist is an opportunity to be exposed to a man and to refute a maladaptive and generalised cognition (i.e. all men are dangerous).

In the case of Olga, she reported:

Olga ‘I think, strange for me is that uhm, therapist and the guy who raped me look very similar …’

Interviewer ‘Wow …’

Olga ‘But, uhm, I never felt unsafe, like you understand?’

The therapist aimed to create a supportive and trusting therapeutic engagement to allow participants to process traumatic experiences. Olga reported that she felt ‘safe’ in treatment and that this provided her an opportunity for a corrective experience with a therapist who, to some extent, and only based on physical appearance, could have evoked difficult emotions and thoughts for her. She chose to engage with this atypical encounter of exposure, stating, ‘… I think because I made that conscious divide between “he’s not him”’. Olga chose to adopt a realistic approach in processing her experiences of traumatic stress.

Participants’ reflection on their engagement with their therapist focused on the nature and quality of the interaction. For example, Thembi reported, ‘… he was engaged. It was not just I am listening to a story because I’m writing a paper, or I have to capture results’. Thembi’s comments suggest the presence of a therapeutic alliance between herself and DB.

The importance of being attentive, interested and genuine in therapy was also reflected in Buyiswa’s experience of therapy. She reported that she told her therapist, ‘I dread coming here and I do not like it’. In turn, the therapist stated, ‘Well, I’m thankful you’re here’. These quotes reflect a sense of honesty and appreciation in treatment, even though participants were simultaneously processing their personal traumas. Furthermore, Fiona reflected on how she experienced the gender difference:

‘[…*A*] very comfortable, very open space to be in. And I think that, his approach, his tone, body language, boundaries being maintained within the space was very helpful in me understanding that this is a safe space.’

For Fiona, the experience of the therapist assisted her in feeling safe and comfortable during the intervention. Her willingness to engage allowed her to experience safety and to be open and reflective, regardless of the gender difference. The experience of gender in trauma therapy illustrates one of the more nuanced challenges that could be encountered regarding gender in trauma therapy.

### Exposure

For most of the participants, exposure was initially, and sometimes throughout treatment, a challenging experience. Thando, who engaged in an *in vivo* activity, initially felt overwhelmed but managed to complete the task. She stated that, ‘it was much, much easier, like the walk itself, there wasn’t any like panic attacks, I was hyper aware, but it was still doable’. For Thando, completing the task provided her with a different experience and insight related to her perceptions of exposure and her ability to engage with feared situations related to her trauma.

Thembi stated that she experienced a sense of disbelief when she realised that, as part of the therapy, she was required to engage with trauma memories she feared and avoided. Thembi stated, ‘In my head, when he was first explaining, I kept thinking “are we really going to deal with my issue here?”’ Thembi’s initial apprehension and disbelief might suggest that trauma survivors enter therapy with some degree of ambivalence about how they would engage with their psychological distress. She referred to how PE was explained to her, which allowed her to understand what was expected and how exposure was to be conducted. She stated, ‘I was like “oh, I get it”’. Her experience of being informed about PE and the rationale for exposure appeared to have made the process more understandable and tolerable.

Even so, engaging in exposure resulted in participants experiencing indecisive moments of commitment to PE and a desire to disengage from therapy. Fiona engaged in imaginal exposure but reported feeling overwhelmed thinking about her experience of sexual assault as a child. Fiona ‘found a way that made it work for me, so instead of just coming there and telling him, I went and then I wrote it down and then I came and read it and it was easier to talk’. Fiona’s experience is an example of how exposure can be challenging for some and that participants needed to understand its purpose and feel safe enough during recounting the traumatic event.

In addition to experiences of disbelief and finding adjusted methods to engage in exposure, participants reported experiences of subtle avoidance. Tessa reported that whilst doing exposure, she found the imaginal exposure exercise difficult and stated she was unsure that she wanted to continue treatment:

Interviewer: ‘Did it ever feel like you were not keen to come back?

Tessa: ‘A few times, yes. [*laugh*] Yeah, especially because we did a lot of recounting, so … the last couple of sessions it felt like “ugh, I’m just talking about the same thing over-and-over again.”’

Tessa stated that she felt frustrated by repeatedly talking about her experiences of rape in several sessions. She felt that the recounting was unpleasant at certain times, stating, ‘In the moment it’s not nice’. Her honest description reflects what other participants probably experienced, which is that exposure can be an unpleasant experience. As part of an *in vivo* activity, Buyiswa was asked to wear the jacket she wore on the day of the attack. She stated:

‘It was hard. I could not get my arms inside the jacket or put it over my shoulders for I think three min. I was like okay; we will get the arms in tomorrow. We will put it away and yeah. Eventually [*I*] got to wear the jacket and it felt good.’

Buyiswa’s experience with her jacket also demonstrates that participants required some time and encouragement in PE. The difficulty of exposure therapy may have impacted her in several ways, which may have led her to feeling overwhelmed, frustrated and needing to disengage. These needs may have been because of the increased levels of distress associated with exposure. Yet participants, amidst the difficulty, also appeared to realise that the fear of exposure was not as threatening or even real as it might have felt, which allowed for the processing of their traumatic memories.

### Experience of recovery

Participants stated that they benefitted in various ways. For example, Buyiswa spoke about how treatment changed her outlook on life and that she continuously attempted to integrate her trauma as part of who she is. She stated, ‘I do not want to say it does not have a hold on me anymore, but it does not have as much hold on me as it did, sadly, [*it is*] part of who I am now’. Buyiswa was able to live a more ‘positive’ life towards the future. Relatedly, Tessa highlighted an important aspect about her experience, namely that she became aware of her own progress during the intervention:

Interviewer ‘What made you come back?’

Tessa ‘I noticed changes, I noticed differences. I thought, it is helping. So, let me just come back.’

Interviewer ‘Do you mind if I ask what changes you noticed?’

Tessa ‘I am dealing with things. I am not just repressing them and expecting them to go away. I am not avoiding the emotions that I felt in those situations and what I feel thinking back to what happened. I am thinking about it and engaging with it and, trying to understand how to move on …’

Importantly, Tessa noticed that she showed less avoidance regarding her trauma-related fears, which proved to be more beneficial compared to dropping out of treatment. Her experience also highlights how participants need to regulate the discomfort during treatment. If this is successfully achieved, participants may gradually experience increments of progress during and/or after treatment.

For Thembi, she tended to self-isolate and did not know how to accept support from others. However, she decided to commit to the process of the treatment, stating ‘that any good programme can be put in place … But if someone is not like allowing themselves to fully engage in something, then it’s never going to happen …’. Thembi alluded to the active and reciprocal nature of PE. She stated:

‘The first session I really did not want to be there, but I realised I needed to be there. Me needing to be there outweighed me not wanting to be there. So, I sat, and I was honest, and I was as open as I could be, because I realised the end goal.’

Thembi’s decision to commit to treatment enabled her to engage with the difficult aspects of her trauma. Her decision to go against her tendency of avoidance provided her with the opportunity to process her experiences of trauma.

## Discussion

The broad aim of this article was to explore the perceptions and experiences of trauma survivors receiving PE for PTSD and to ascertain if the qualitative findings would suggest that PE is an acceptable trauma therapy in a LMIC. Five themes emerged from our data that relate to how participants perceived and experienced PE as a trauma therapy for PTSD.

Firstly, some participants expressed an appreciation for the structured nature of PE. Importantly, the experience of a structured treatment approach defuses the critique against manualised psychotherapies as limiting and imposing a ‘power’ imbalance on the treatment process and the client.^16^ Furthermore, research on treatment drop-out rates found that nonstructured therapies, among others, had a higher drop-out rate compared to manualised treatments.^9,17^ Yet the findings also suggested that participants’ avoidance and frustration with the structure of treatment needed to be managed in a responsive manner. Hembree et al.^14^ state that avoidance during treatment is a major obstacle which requires honest and supportive engagement during treatment.

Secondly, two participants reflected on obstacles related to limited empathy and being frustrated with the structure of treatment. For example, one participant reflected on how she wanted the therapist to be more reassuring and empathetic towards her experience of rape. Another participant felt frustrated because she wanted a quick answer to her complex history of psychological trauma. Arguably, these reflections can be related to therapeutic alliance difficulties in therapy. Therapeutic alliance is generally described as the bond between the therapist and client and is characterised by various factors such as empathic resonance, goal and task agreement, mutual affirmation and commitment to treatment, among others.^18,19^

McLaughlin et al.^18^ investigated therapeutic alliance difficulties (i.e. rupture repair or U-shaped alliance) in PE treatment for PTSD. In a clinically representative sample (*n* = 116), the authors found that rupture repair was common, and that therapists need to be cognisant of repair to enhance treatment outcomes.^18^ Moreover, McLaughlin et al. highlighted that persons with comorbid depression and childhood abuse might have more variability in establishing a therapeutic alliance. Arguably, the obstacles experienced by Anna and Tessa are understood as a process of rupture repair, which is common, and that their comorbid depression and histories childhood abuse further compounded the process of establishing a therapeutic alliance.^18^

Thirdly, aspects related to gender difference between therapist and some participants emerged from the data. Literature on gender matching in psychotherapy for PTSD is sparse, but notions of personal characteristic matching have existed for several decades.^20,21^ Shiner et al.^22^ found that gender match in psychotherapy between female trauma survivors and female therapists was not associated with treatment retention and that gender preferences appeared to be arbitrary.^22^ Rather, participants reported to have had a sense of safety in treatment, which enabled them to engage in and complete the treatment. Thus, it would appear, based on the data in this study, that gender difference between participant and therapist had no impact on how participants experienced and/or completed the treatment. Again, it can be suggested that therapeutic alliance, regardless of gender disparity, is a more accurate measure of treatment acceptability.^18^ Yet reflecting on gender differences, especially in trauma therapy, can allay certain anxieties and potentially strengthen the therapeutic alliance, thus enhancing the outcome of treatment.^23^

Fourthly, a misconception of exposure therapies is that they are more technique driven and devoid of therapeutic factors such as empathy, trust and safety.^23^ For example, Buyiswa reported that she felt she could be honest with the therapist and even let him know that she was reluctant to be in therapy but that she also felt supported throughout the treatment process. Sherwood^21^ found that recovery in therapy is when clients feel safe and supported in therapy, which is what is reflected in Buyiswa’s statement. Moreover, some scholars of psychotherapy have emphasised the relational process of treatment, yet in this study, it can be argued that a combination of clear intervention technique (i.e. exposure strategies) paired with a therapeutic alliance (relational) is necessary.^12,14^

As predicted by emotional processing theory,^24^ participants experienced exposure procedures as distressing.^14^ Participants had wavering experiences of commitment and the desire to drop out of treatment. Exposure appeared to be a primary reason for potential drop-out, yet participants reported that they had started experiencing a sense of recovery and, therefore, they opted to complete treatment. Several studies have found that participants are expected to experience transient levels of distress at the start of exposure and that no statistically significant iatrogenic outcomes are associated with PE.^25^ Fifthly, participants’ experiences of recovery are reflective of the effectiveness of PE as a treatment for PTSD. Participants did not merely experience symptom improvement but also a sense of reclaiming their lives in different ways by having a different outlook on life.

The implementation of PE in settings like South Africa remains limited and has several barriers to consider. For example, structural limitations such as limited health infrastructure and trained professionals can impede the implementation of effective therapies such as PE.^26^ Moreover, on an epistemological and ideological level, critical psychology and cultural relativity perpetuate a parochial dichotomy between empirical psychology (i.e. PE) and what is a so-called relevant psychology (i.e. African psychology) that, arguably, results in a divided approach to addressing social and health issues in society.^27^

To this end, and based on the perceptions and experiences of the participants, the findings of this study contribute to the scant literature on the acceptability of PE for PTSD in South Africa. Importantly, the findings also suggest that persons from South Africa, which is a contextually and culturally diverse nation, perceived and experienced PE as a somewhat challenging and also beneficial therapy for the treatment of PTSD.

### Limitations

The findings of the study have some limitations. Firstly, the all-female sample and level of education (tertiary) of the participants might limit the transportability of findings to persons with a lower level of education and who are male. It is also worth noting that a brief PE version was used and not the standard PE; therefore, our findings cannot be applied to standard PE. Thus, further research using a more heterogenous sample (i.e. gender) and standard PE would add further insight regarding the acceptability of PE in a South African context.

## Conclusion

The findings of the article are in keeping with the extant literature on PE. The textual data suggest that participants found PE to be beneficial for the treatment of PTSD. Moreover, the study suggest that PE is an acceptable and beneficial trauma therapy in a contextually diverse setting such as South Africa. Overall, considering the evidence base of PE for the treatment of PTSD, this study serves as a move towards understanding the acceptability of PE in a South African setting.
